# Permeability Is the Key in Sublingual and Buccal Drug Delivery

**DOI:** 10.3390/pharmaceutics18101268

**Published:** 2026-10-07

**Authors:** Rand Musa Obaid Alfawaz, Hassan Almoazen

**Affiliations:** 1 College of Pharmacy, King Saud bin Abdul Aziz University of Health Sciences (KSAU-HS), Riyadh 11481, Saudi Arabia; alfawazr47061@ksau-hs.edu.sa; 2 Department of Pharmaceutical Sciences, College of Pharmacy, University of Tennessee Health Science Center, 881 Madison Ave, Rm 661, Memphis, TN 38163, USA

## 1. Introduction

Sublingual and buccal drug delivery has evolved from a route reserved for a handful of cardiovascular and analgesic agents into an actively investigated non-invasive delivery platform within pharmaceutical sciences. The oral mucosa’s rich vascularization, avoidance of first-pass hepatic metabolism, and feasibility for self-administration make it an attractive route for low-dose, high-potency therapeutics and an increasingly attractive alternative for vaccines and biologics that would otherwise require injection. Over the past decade, the field has moved beyond simple translational formulations (i.e., fast-dissolving tablets and films) designed for pediatric and geriatric patients who cannot reliably swallow solid doses and shifted toward nanoparticle-based carriers and multilayered mucoadhesive systems.

Despite this momentum, important gaps have persisted in the scientific literature. Mechanistic understanding of how formulation physicochemical properties such as micro-viscosity, muco-adhesion, and permeability translate into therapeutic outcomes has often lagged behind formulation innovation itself. Clinical validation beyond preclinical and ex vivo models remains comparatively limited, and the protective, barrier-reinforcing side of mucosal formulation science has received far less attention than permeation enhancement. This Special Issue was assembled to address some of these gaps, and the five contributions collected here, spanning vaccine delivery, periodontal therapy, antifungal formulation, and mucosal barrier engineering, offer a representative view of where the field currently stands and where meaningful research work remains to be done. This closing editorial summarizes those contributions, situates them within the broader trajectory of the field, and outlines the research directions that we believe deserve the greatest attention going forward.

## 2. Overview of Published Work

The five papers collected in this Special Issue span a broad range of questions relevant to sublingual and buccal drug delivery, from vaccine formulation to clinical validation, antifungal therapy, mucosal barrier engineering, and the historical trajectory of the field itself. Sublingual and buccal delivery has long been valued for its combination of high vascularization, avoidance of first-pass hepatic metabolism, and ease of self-administration, but translating these theoretical advantages into reliable clinical products has required continued advances in formulation science, delivery system design, and mechanistic understanding of how mucosal tissue interacts with a given dosage form. Read together, the five contributions trace a field that is maturing along several fronts at once, moving from mechanistic formulation science through real clinical outcomes, while also revealing how much work remains before many of these novel approaches reach patients.

Sublingual vaccination has been explored for decades as a route for mucosal immunization, yet the formulation determinants of a successful response have remained poorly defined. Contribution (1) addresses this gap directly by systematically correlating formulation rheology, mucoadhesion, and in situ mucosal retention with the in vivo immunogenicity of a sublingual model vaccine in mice. Using an ovalbumin-based model antigen, the authors compared HPMC and chitosan-based excipients, tracking mucosal residence by IVIS imaging and measuring both systemic (serum IgG) and mucosal (IgA) antibody responses. Contrary to the common assumption that stronger mucoadhesion improves vaccine performance, the most viscous and adhesive excipient tested, HPMC, produced the weakest immune response, while the less retentive but permeation-enhancing chitosan excipient elicited stronger systemic and mucosal immunity, even at reduced antigen and adjuvant doses. This finding reframes the design question for sublingual vaccines around balancing adequate retention with efficient antigen release and uptake, rather than maximizing adhesion alone, and offers a useful mechanistic caution for the growing field of needle-free vaccine design [1].

Moving from systemic immunization to local disease management, Contribution (2) evaluated metronidazole and metronidazole benzoate loaded gel and film formulations as adjuncts to standard scaling and root planing (SRP) therapy in a randomized, double blind clinical trial involving forty-five patients with stage I to II periodontitis. Patients were divided into three groups, SRP alone, SRP plus film, and SRP plus gel, with outcomes assessed longitudinally through probing depth, clinical attachment level, and bleeding on probing, alongside HPLC-based quantification of drug concentration in gingival crevicular fluid. Both formulations improved clinical outcomes compared with conventional treatment alone, with the gel formulation producing the greatest improvement in clinical attachment level, while drug concentrations did not differ statistically between the two delivery formats. This contribution offered direct clinical validation of a mucoadhesive local therapy in real patients, distinguishing it from the preclinical and formulation stage work that characterizes the rest of the issue, and demonstrating that buccal and periodontal drug delivery can reduce reliance on systemic antibiotic exposure [2].

Formulation optimization through rigorous experimental design was exemplified in Contribution (3), which developed a miconazole-loaded microemulsion for buccal treatment of oral candidiasis using a full factorial design. Using oleic acid as the lipophilic phase and Tween 20 and PEG 400 stabilizer systems, the authors systematically varied three formulation variables and characterized the resulting systems by pH, conductivity, refractive index, droplet size, zeta potential, rheology, and goniometry. The optimized formulation, containing 2% miconazole base, 5% oleic acid, 40% Tween 20, and 20% PEG 400, achieved a mean droplet size of approximately 120 nanometers, a work of adhesion of 71.98 mN/m, and a favorable diffusion coefficient, alongside two promising gel-like variants. This contribution stands as an instructive template for rational, statistically driven formulation design, illustrating how systematic modeling rather than trial and error can balance droplet size, mucoadhesive strength, and drug diffusion for locally acting buccal therapy [3].

Contribution (4) approached mucosal delivery from the opposite direction: rather than enhancing permeation, it engineered hydrophobic, hydrophilic, and liposomal formulations composed of lipids resembling those in the skin’s stratum corneum, designed to reduce transmucosal permeability. Tested against caffeine, ibuprofen, dexamethasone, and ivermectin across porcine skin, native mucosa, and treated mucosa, the most effective liposomal formulation reduced permeability coefficients by 40 to 80%, particularly for low molecular weight compounds. This “waterproofing” strategy highlights an underexplored application of mucosal formulation science, protecting the oral barrier against toxins, contaminants, and pathogens, including viruses, rather than only facilitating drug absorption. It opened a genuinely novel research direction that complemented the permeation-enhancing focus of the rest of the issue, reframing mucosal permeability as a property that can be modulated in either direction depending on the therapeutic goal [4].

Finally, Contribution (5) is a historical review co-authored by the Special Issue’s Guest Editor, which spans the sublingual and buccal contributions within 75 years of the field’s development. It traced four successive growth phases in the field’s publication history and identified the current era as one shaped by nanoparticle-based carriers, multilayered mucoadhesive delivery systems, pediatric- and geriatric-friendly fast-dissolving formulations, and a renewed interest in delivering vaccines and biologics such as peptides and proteins through the sublingual and buccal routes. The review highlights the oral mucosa’s inherent advantages, including high vascularization, avoidance of first-pass hepatic metabolism, and ease of self-administration, as the enduring rationale for the field’s continued growth, while identifying large, unmet gaps in delivering macromolecules such as peptides, proteins, and nucleic acids through this route [5].

Combined together, these five contributions illustrate the field’s progress along complementary axes: mechanistic understanding of the relationship between formulation properties and immunogenicity, clinical translation of local buccal therapy, rational and statistically grounded formulation design, an emerging barrier engineering perspective, and a historical framework that ties the field’s past to its future direction. Each contribution addresses a distinct piece of the same underlying challenge: how to design a mucosal formulation that reliably achieves its intended biological outcome, whether that outcome is immune activation, local antimicrobial or antifungal action, or protection of the tissue itself, and together they set the stage for the priorities discussed in the following section.

## 3. Future Directions

Enhancing drug permeability continues to be one of the pharmaceutical sciences’ primary focus areas into the twenty-first century. The wealth of knowledge we have currently achieved in cellular biology and medicinal chemistry allows us to intelligently work on figuring out new mechanisms to enhance penetration through tight junctions (paracellular) and lipid membranes (transcellular). Several techniques have been proposed, ranging from tight junction modulators, lipid membrane fluidization, membrane perturbation, drug complexation or ion pairing, extracellular calcium chelation, efflux transporter modulation, and other mechanisms. The use of AI certainly will accelerate discovery in this area by enhancing the design of new penetration enhancers that function on paracellular and transcellular targets. Considering the use of microneedles as a new modality to enhance the penetration of large molecules into skin, we anticipate seeing more applications of microneedles as penetration enhancers into tissues that have not utilized microneedles, such as sublingual and buccal delivery routes. Another area that may attract attention is restricting penetration to achieve a sustained release effect over a longer time span. There are no limits to creativity that will certainly impact this area of research in biology and chemistry [6,7].
